# Association of serum uric acid levels with suicide risk in female patients with major depressive disorder: a comparative cross-sectional study

**DOI:** 10.1186/s12888-020-02891-8

**Published:** 2020-09-29

**Authors:** Jing-Xu Chen, Jun-Hui Feng, Li-Gang Zhang, Yan Liu, Fu-De Yang, Shao-Li Wang, Yun-Long Tan, Yun-Ai Su

**Affiliations:** 1 Beijing HuiLongGuan Hospital, Peking University HuiLongGuan Clinical Medical School, Beijing, China; 2 Jining Psychiatric Hospital, Jining, Shandong Province China; 3 The Department of Psychiatry of Shengli Hospital, Sinopec Shengli Petroleum Administration, Dongying, Shandong Province China; 4 Peking University Sixth Hospital, Peking University Institute of Mental Health, NHC Key Laboratory of Mental Health (Peking University), National Clinical Research Center for Mental Disorders (Peking University Sixth Hospital), Beijing, China

**Keywords:** Uric acid, Suicide risk, Major depressive disorder, Female

## Abstract

**Background:**

Individuals with major depressive disorder (MDD) have a high suicide risk. Some evidence suggests that uric acid (UA) may be involved in the pathophysiology of MDD. The purpose of this study was to evaluate whether serum UA levels were associated with suicide risk in MDD patients.

**Methods:**

One hundred four female patients with MDD (52 patients with suicide risk and 52 patients without suicide risk) and 52 healthy individuals were included in this study. The suicide risk was evaluated by Mini International Neuropsychiatric Interview (M.I.N.I.). Fasting serum levels of UA, as well as glucose, lipid and renal function indicators were measured.

**Results:**

Serum UA levels in MDD patients with suicide risk (245.01 ± 55.44 μmol/L) were significantly lower than those in MDD patients without suicide risk (274.17 ± 72.65 μmol/L) (*p* = 0.017) and healthy controls (271.42 ± 55.25 μmol/L) (*p* = 0.030). There was no difference in serum UA levels between the MDD patients without suicide risk and healthy controls (*p* = 0.821). Binary logistic regression analysis revealed a significant relationship between suicide risk and decreased serum UA levels (OR = 0.989, *p* = 0.010) in MDD patients.

**Conclusion:**

Decreased serum UA levels were associated with suicide risk in MDD patients. Purinergic system dysfunction may be involved in the neurobiological basis of suicide risk in these patients.

## Background

Major depressive disorder (MDD) is one of the most common and serious neuropsychiatric disorders with a current prevalence rate of approximately 2.2% in men and 3.3% in women in the general population of China [1]. Among individuals suffering from MDD, an estimated 15% have suicide ideation while as many as 6% is attempt suicide [2]. Most of the individuals with suicide attempts may meet the diagnostic criteria for MDD [3]. Although there are several dedicated suicide risk assessment instruments in clinical practice, such as Beck Hopelessness Scale and Suicide Ideation Scale, it is very difficult to predict and distinguish the individuals who are vulnerable to suicide [4]. An improved understanding of the pathophysiology of suicide risk in patients with MDD is critical for developing specific targeted strategies for prevention and intervention.

Strong evidence has indicated that there are biochemical and morphological abnormalities in certain brain regions of MDD patients and suicide victims [5–7]. The purinergic system, which includes not only extracellular nucleosides and nucleotides, such as adenosine and adenosine triphosphate (ATP), but also various receptor subtypes, such as adenosine A1 and A2A receptors, is widely expressed in the central nervous system (CNS) [8]. Several experimental studies have indicated that dysregulation of the purinergic system might play an important role in the pathophysiology of suicide [9–12] .

Uric acid (UA), as an easily detectable marker, is the final compound generated from the catabolism of purines in humans [13]. As a powerful endogenous antioxidant, UA accounts for more than 60% of free radical scavenging capacity in human blood [14, 15]. Notably, the CNS is considered to be one of the main sites of UA antioxidation [16]. Recently, numerous clinical data suggest that UA might be one of the potential contributing factors in behavioural and neuronal responses. For example, abnormal serum UA levels have been reported in individuals with cognitive dysfunction, dementia, schizophrenia, bipolar disorder, and sleep disorder [17–20]. The potential involvement of oxidative/antioxidant function in MDD has been demonstrated [21]. Unfortunately, investigations about the causal relationship between UA and MDD have produced inconsistent results and there is still no conclusive evidence [22–24]. To date, few studies have been designed to explore the association between UA and suicide. Recently, promising findings from Bartoli and his team have shown an inverse relationship between UA levels and severity of suicidal ideation in individuals with major affective disorders [25, 26] . However, some confounding factors such as glucose and lipid metabolism, renal function and prescribed medications were not controlled in these studies.

In consideration of the relationship between UA levels and MDD or suicide risk remains poorly understood, we carried out this study aimed at investigating and comparing serum UA levels in MDD patients with or without suicide risk. It is well known that females are more likely to suffer from depression and to attempt suicide [27, 28] than males, and sex is one of the most important factors affecting serum UA levels. Previous evidence indicates that UA levels in men are higher than those in women in both the normal population and MDD patients [26]. Moreover, antidepressant treatment may significantly affect serum UA levels [29]. Therefore, only untreated female subjects were allowed to participate in the present study. Based on existing data, we generated the following hypotheses: 1) serum UA levels in the patient groups would be significantly lower than those in the healthy control group [2]; there would be a significant relationship between decreased serum UA levels and suicide risk in patients with MDD.

## Methods

### Subjects

All patients were consecutively recruited from January 2016 to December 2017 in Beijing HuiLongGuan Hospital, a major psychiatric hospital with over 1000 psychiatric hospital beds in Beijing, China. The study was approved by the Ethics Committee of Beijing HuiLongGuan Hospital, and all participants provided written informed consent in accordance with National Health and Medical Research Council guidelines.

Eligible newly admitted patients were female, aged 18–65 years, Han ethnicity, non-medicated in the past month, and had a Diagnostic and Statistical Manual of Mental Disorders, fourth edition (DSM-IV) diagnosis of MDD, the scores of 17 item Hamilton Depression Rating Scale (HAMD-17) ≥17. The exclusion criteria were as follows: (1) patients in a remission state; (2) patients with other diagnoses such as organic mental disorders, alcohol use disorders, psychotic disorders, mental retardation, neurological illness, renal disease and endocrinal dysfunction or another medical illness which would affect UA levels; and (3) patients who had undergone treatment with electroconvulsive therapy (ECT) within the last month. (4) being pregnant or breastfeeding.

Patients with suicide risk were classified as the suicide risk group and those without suicide risk were classified as the non-suicide risk group. Both were age-matched and selected from the whole eligible patients. In addition, the control group of healthy individuals matched for age to patient groups were enrolled; healthy participants included Han Chinese individuals without a personal or a positive family history for any mental illness, who were recruited from the community near the hospital. Neither patients nor healthy subjects presented drug or alcohol abuse/dependence, pregnancy or breastfeeding, or the use of any medication that may have influenced UA levels. Smoking has been shown to affect UA levels [30], so smokers were excluded from the study. This was confirmed based on the criterion: a smoker was an individual who smoked either daily or occasionally in the 1 month preceding the time of survey.

### Mental-status assessment

The DSM-IV diagnosis of MDD was established according to the Chinese version of Mini International Neuropsychiatric Interview (M.I.N.I.), Version 5.0 [31, 32]. All patients were native in the language of interview. M.I.N.I. is a short-structured interview that consists of six questions about past and current suicidality. Scores of the suicide risk section ranged from 0 to 33 and were classified into four subgroups: 0 score index, non-suicide risk; 1–5 score index, low suicide risk; 6–9 score index, medium suicide risk; and ≥ 10 score index, high suicide risk. In this study, all the patients with MDD were classified as having an absence (low or none) or presence of suicide risk (moderate or high) based on previous studies [32–34]. Severity of depression symptoms were assessed by the 17 item Hamilton Depression Rating Scale (HAMD-17). Item 3 (suicide) was excluded when we evaluated the association between suicide risk and severity of depression. To ensure consistency and reliability of ratings across the study, four psychiatrists with over 5 years of experiences in clinical practice simultaneously attended a training session on the use of the M.I.N.I. and HAMD-17 before starting this study.

### Biochemical measurements

Blood samples (5 ml) were taken from all the subjects by venepuncture and collected into an anticoagulant-free vacuum tube between the hours of 6:00 AM and 7:00 AM after a 12 h overnight fast. The blood was centrifuged at 4000 g for 10 min within 1 h, and resulting serum was withdrawn and analysed immediately. Serum UA levels were measured with a sandwich enzyme-linked immunosorbent assay (ELISA) using a commercially available kit (DRG kit Cat#: EIA-2925, Germany). Each sample was run in duplicate. All the samples were performed a quality analysis and assayed by the same technician, who was blinded to the clinical situation. The sensitivity was 0.25 μmol/L, and the intra-assay and inter-assay variation coefficients were 2.1 and 2.7%, respectively. We also collected blood glucose, lipid metabolism and kidney function indicators, which are considered potential confounders of serum UA levels [35, 36].

### Statistical analysis

All the continuous variables are summarized as means and standard deviation or medians and interquartile ranges, as appropriate. The categorical variables are presented as raw numbers and percentages (%). For the comparison of the continuous variables among groups, we used one-way ANOVA or independent sample t-tests for parametric variables and the Mann-Whitney test for non-parametric variables. When significant differences were detected by ANOVA, post hoc multiple comparison analyses with Bonferroni adjustment were performed. In addition, UA levels and total cholesterol were compared among groups after controlling for the potentially confounding effects of variables that significantly differed among groups in univariate analyses using analysis of covariance (ANCOVA). A binary logistic regression model was used for the multivariate analysis. Odds ratios (ORs) and 95% confidence intervals (CIs) were calculated. All statistical tests were two tailed and a *p*-value < 0.05 was considered statistically significant. SPSS (Ver. 17.0) was used for the statistical analyses.

## Results

A total of 198 eligible patients suffering from MDD were enrolled, of which 52 patients (26.3%) presented a risk of suicide. Two patients who were smokers were excluded. Control data of 52 MDD patients without suicide risk and 52 healthy subjects were included in this study. The demographic characteristics of healthy controls and patients with MDD are presented in Table 1. Among the three groups, there were significant differences in marital status (χ^**2**^ = 9.424, *p* = 0.009), employment rates (χ^**2**^ = 6.721, *p* = 0.035) and BMI (F = 3.408, *p* = 0.036), but not in education (*p* > 0.05). Among the subjects with MDD, the proportion of married subjects in the suicide risk group was lower than that in the non-suicide risk group (χ^**2**^ = 5.786, *p* = 0.016). As shown in Table 2, there were no statistically significant differences between the two patient groups in most of their clinical characteristics including the age of onset, number of episodes, duration of illness, rate of family history of psychiatric disorder and first-episode of depression (all *p* > 0.05), while HAMD-17 total score in the suicide risk group was higher than that in the non-suicide group (t = 4.423, *p* < 0.001).

**Table 1:** Demographic characteristics of healthy controls and patients with MDD

	Healthy controls (*N* = 52)	Patients without suicide risk (*N* = 52)	Patients with suicide risk (*N* = 52)	F/χ^2^	df	*p*
Age (years)	44.44 (11.60)	44.31 (11.72)	44.38 (11.65)	0.002	153	0.998
Education (years)	12.63 (5.09)	12.83 (4.76)	12.33 (4.90)	0.135	153	0.874
Married, n (%)	44 (84.6)	43 (82.7)	32 (61.5)^ab^	9.424	2	0.009
Employed, n (%)	43 (82.7)	36 (69.2)	31 (59.6)^a^	6.721	2	0.035
BMI (kg/m^2^)	22.95 (2.79)	22.10 (2.54) ^a^	21.62 (2.68) ^a^	3.333	153	0.038

Data are mean ± SD unless otherwise indicated. a, compared with healthy controls, *p* < 0.05; b, compared with patients without suicide risk, *p* < 0.05

**Table 2:** Clinical characteristics of depressive subjects with or without suicide risk

	Patients without suicide risk (*N* = 52)	Patients with suicide risk (*N* = 52)	t/χ^2^/Z	df	*p*
Age at onset (years)	38.85 (12.58)	35.71 (11.36)	3.028	102	0.185
HAMD-17 score*	20.63 (2.95)	23.63 (3.90)	4.423	102	< 0.001
MDD, single episode, n (%)	16 (30.8)	23 (44.2)	2.010	2	0.156
Family history of psychiatric disorder, n (%)	15 (28.8)	12 (23.1)	0.450	2	0.502
Number of episodes, median (P_25_, P_75_)	2 (1, 3)	22 (1, 4)	0.220	---^a^	0.826
Duration of illness (Months), median (P_25_, P_75_)	33.0 (12.5, 103.5)	38.5 (4.9, 218.3)	0.133	---^a^	0.894
Scores of suicide risk, median (P_25_, P_75_)	1 (0, 3)	11 (9, 19)	8.859	---^a^	< 0.001

Data are means ± SD unless otherwise indicated. HMAD-17:17 item Hamilton Depression Rating Scale. a, Mann–Whitney U-test. *Item 3 (suicide) was excluded from the total score

Table 3 shows the routine biochemical markers including total cholesterol (CHO), triglycerides (TG), high-density lipoprotein cholesterol (HDL-C), low-density lipoprotein cholesterol (LDL-C), fasting blood glucose (FBG), creatinine, urea and UA in the three groups. Significant differences were found only in the serum levels of CHO (F = 3.219, *p* = 0.043), creatinine (F = 4.577, *p* = 0.012) and UA (F = 3.546, *p* = 0.031). Post hoc analyses showed that the CHO levels between the two control groups were not significantly different (*p* > 0.05), whereas both were higher than those in the patients with suicide risk group (patients without suicide risk: *p* = 0.045; healthy controls: *p* = 0.021). Creatinine levels were significantly lower in both patient groups when compared with healthy individuals (patients without suicide risk: *p* = 0.005; patients with suicide risk: *p* = 0.020); however, no significant differences were detected between the two patient groups (*p* > 0.05). Serum UA levels were significantly lower in patients with suicide risk than in patients without suicide risk (*p* = 0.017) and healthy individuals (*p* = 0.030), while there were no differences between the two latter groups (*p* = 0.852).

**Table 3:** Biochemical indices of blood serum in healthy controls and patients with MDD

	Healthy controls (N = 52)	Patients without suicide risk (N = 52)	Patients with suicide risk (N = 52)	F	df	*p*
FBG (mmol/L)	4.81 (0.70)	4.93 (1.03)	5.01 (1.30)	0.490	153	0.613
TG (mmol/L)	1.38 (0.61)	1.29 (0.63)	1.25 (0.66)	0.626	153	0.536
CHO (mmol/L)	4.31 (0.99)	4.26 (0.95)	3.88 (0.86)^ab^	3.219	153	0.043
LDL-C (mmol/L)	2.43 (1.00)	2.47 (0.87)	2.29 (0.83)	0.550	153	0.584
HDL-C (mmol/L)	1.31 (0.30)	1.23 (0.36)	1.35 (0.43)	1.297	153	0.277
Urea (mmol/L)	4.58 (1.06)	4.32 (1.53)	4.24 (1.11)	1.065	153	0.347
Creatinine (μmol/L)	66.51 (11.04)	60.4 (11.40) ^a^	61.43 (10.59)^a^	4.577	153	0.012
UA (μmol/L)	271.42 (55.25)	274.17 (72.65)	245.01 (55.44)^ab^	3.546	153	0.031

Data are means ± SD unless otherwise indicated. *FBG* fasting blood glucose; *CHO* total cholesterol, *TG* triglyceride, *HDL-C* high density lipoprotein cholesterol; *LDL-C* low density lipoprotein cholesterol

^a^compared with healthy controls, *p* < 0.05

^b^compared with patients without suicide risk, *p* < 0.05

Analysis of covariance showed that serum UA level differences among the three groups remained statistically significant after adjusting for marital status, employment status and BMI (F _(5,150)_ = 3.709, *p* = 0.027). Further pairwise comparisons indicated that patients with suicide risk had lower serum UA levels than patients without suicide risk (*p* = 0.010) and healthy individuals (*p* = 0.046), respectively. However, there was no significant difference with respect to serum levels of CHO among the three groups after adjusting for confounding factors (F _(5,150)_ = 2.731, *p* = 0.068).

The results of the binary logistic regression analysis are listed in Table 4. We used the significant independent variables from the comparisons between patients with suicide risk and patients without suicide risk including marital status, the levels of CHO, HAMD-17 total score and UA. This regression analysis showed that unmarried status, more severe depressive symptoms, decreased levels of UA increased the odds of suicide risk.

**Table 4:** Factors associated with suicide risk in MDD patients: multivariate Logistic regression analysis

Variables	Coefficient β	OR	95%CI		*p*
			Lower	Upper	
Marital status (Married)	−1.583	0.205	0.061	0.696	0.011
HAMD-17 total score*	0.239	1.270	1.105	1.459	0.001
UA	−0.011	0.989	0.983	0.998	0.019

*UA* uric acid; *HMAD-17* 17 item Hamilton Depression Rating Scale. *Item 3 (suicide) was excluded from the total score

## Discussion

The principal finding of this study was that serum UA levels among MDD subjects with suicide risk were significantly lower than those in patients without suicide risk, indicating possible decreased function of the purinergic system-in comparison with MDD patients without suicide risk. It could be inferred that serum UA levels in the CNS of those patients with suicide risk were also reduced, since there was a positive linear correlation between peripheral and central UA levels [16]. In this study, we also found that being unmarried was a risk factor for suicide, consistent with the results of previous studies in the general population [37].

To the best of our knowledge, this study is first to assess the association between the concentration of serum UA and suicide risk in drug-naïve patients with MDD. Of note, the sample of our comparative cross-sectional study consisted of only female subjects and included an appropriate group of depressive patients without suicide risk and healthy individuals. Moreover, several potential confounders, such as variables associated with metabolism and renal function, were taken into account. Specifically, previous evidence showed altered UA levels are influenced by lipid metabolism [38], univariate analysis without adjusting for confounding factors, showed significant differences of serum levels of CHO existed between patients with suicide risk and patients without suicide risk. However, further analysis imply it is not an independent risk factor for suicide risk. On the other hand, there is also evidence that the possible interaction exists between UA metabolism and lipid metabolism [39]. Further investigation is warranted to validate this finding. Our results were in general agreement with previous research that revealed an inverse relationship between UA levels and suicidal ideation severity in patients with major affective disorders [25, 26]. In addition, a decrease in the output of UA was also found in the urine of nonchemical suicide victims, although only 12 patients were included [40]. Based on the available data, we speculate from our results that UA levels might act as a candidate biological marker to discriminate MDD patients with and without suicide risk.

Several hypotheses were proposed to explain the biological mechanism by which low UA levels may influence suicide risk, although the mechanism is far from clear. The first theory is related to the antioxidant properties of UA. Low UA levels lead to the overproduction of free radicals which in turn may cause oxidative damage to brain neuron integrity and function. In fact, oxidative stress is a notorious major underlying mechanism responsible for suicide independent of the effects of depression and classical risk factors, such as marital status [41]. Second, low UA levels imply that other purine metabolism may also be dysregulated, which might contribute to suicide risk. Adenosine, a purine cycle nucleoside, is an important endogenous inhibitory neuromodulator. The activation of adenosine A1 receptors inhibits neurotransmitter release such as glutamate release and suppresses the production of pro-inflammatory cytokines [42, 43]. Therefore, UA may play an important role in neuroregeneration and neuroprotection [42], while neurodegeneration and neuroplasticity have been suggested as part of the neurobiology of suicide [6, 42].

Over the past few years, decreased UA levels have been reported in individuals with current, but not remitted, MDD of both sexes and could be reversed after treatment with certain antidepressant drugs [22, 24, 44, 45]. Moreover, UA levels in patients presenting MDD were also significantly lower than those in patients presenting other mental disorders such as dementia, substance-related disorders, schizophrenia and bipolar disorder [24, 46]. In contrast, some similar research did not replicate these findings [47–49], even yielding opposite results [23]. Notably, the present study showed that serum UA levels significantly decreased in only MDD patients with suicide risk, and not in those without suicide risk. More recent evidence indicates that UA levels have a strong negative association with the severity of depressive symptoms [44, 50], while depression severity is one of most important risk factors for suicide [51, 52].

Thus, we speculated that the inconsistency with previous research might be related to serum UA levels specifically decreasing due to severe MMD. Another possible explanation for the discrepancy is the high heterogeneity between studies, especially between the sample demographics and clinical characteristics, such as age and BMI, which can significantly influence UA levels.

Several limitations of the present study are worth mentioning. First, in this cross-sectional study with a relatively small number of female subjects, clinical subtypes of MDD (such as psychotic, atypical or melancholic features and seasonal onset) were not considered- all of which may limit the generalization of findings. Our results need replication in a larger sample study with additional confounding factors considered and both male and female patients included. Second, although unified dietary patterns were provided in inpatients and healthy individuals were advised to consume a light diet the whole day before blood samples were drawn, the amount and type of dietary were possibly different among the groups. Third, any altered metabolic activity of purinergic system may be linked with abnormal UA levels, and we did not evaluate the levels of more purine metabolites, such as adenosine inosine, xanthine, and hypoxanthine. Additionally, genetic variations, including purinergic gene expression and single nucleotide polymorphisms (SNPs), for instance, *P2X7* gene, have been found to show an association with increased risk for mood disorders [8]. Future studies should measure these related genetic variations and then explore their roles in contributing to abnormal UA levels. Fourth, it has been shown that impulsivity, i.e., one of the main clinical correlates of suicide, may have a positive association with serum UA [53]. However, we did not consider impulsivity levels in our study. Fifth, menstrual and premenopausal hormones affect UA levels, for example, after menopause and at the time of onset of menstruation, the concentration of uric acid in the plasma increases [54]. In our study, the effects of female menstrual and premenopausal hormones on UA levels were not considered. Last, Although M.I.N.I.’s suicidal module has been widely used in research [55–57], other special questionnaires of suicide assessment have been recommended, such as the Columbia–Suicide Severity Rating Scale (C-SSRS) [58].

## Conclusions

Our study provides preliminary evidence of dysregulation of the purine cycle manifested as decreased levels of serum UA in MDD patients with suicide risk compared with those without suicide risk. Our results indicate that the purinergic imbalance might act as a characteristic biological marker of suicide risk in MDD patients, which might increase the better understanding of the physiological mechanisms of suicide and offer a potential target for effective therapeutic interventions and early preventions.

## Data Availability

The datasets used and/or analysed during the current study are available from the corresponding author on reasonable request.
